# *Moringa oleifera* mediated green synthesis of gold nanoparticles and their anti-cancer activity against A549 cell line of lung cancer through ROS/ mitochondrial damage

**DOI:** 10.3389/fchem.2025.1521089

**Published:** 2025-03-05

**Authors:** Dawei Qian, Dongsheng Zha, Yuanyao Sang, Jiangquan Tao, Youshuang Cheng

**Affiliations:** ^1^ Department of Thoracic Surgery, Tongling Yi’an District People’s Hospital, Tongling, Anhui, China; ^2^ Department of Thoracic Surgery, Shanghai Ninth People’s Hospital, Shanghai Jiaotong University, Shanghai, China

**Keywords:** gold nanoparticles, *Moringa oleifera*, anti-cancer, cytotoxicity, mitochondrial damage, ROS production, A549 lung cancer cell line

## Abstract

**Introduction:**

Gold nanoparticles (Au-NPs) hold significant promise in lung cancer treatment due to their unique physicochemical properties, enabling targeted drug delivery, enhanced therapeutic efficacy, and reduced systemic toxicity. This study is aimed to produce the Au-NPs utilising *Moringa oleifera* and evaluate their effectiveness in the treatment of lung cancer, with a specific focus on A549 cell lines.

**Methods:**

The synthesis of Au-NPs was carried out by combining 10 mL of an aqueous extract of M. oleifera with 190 mL of a 1 mM HAuCl4 solution. The synthesized Au-NPs were characterised using several microscopic and spectroscopic techniques. The evaluation of the median inhibitory concentration (IC50) of Au-NPs and its impact on apoptosis was conducted through the measurement of caspase activation and the formation of reactive oxygen species (ROS). Anti-cancer characteristics was conducted by employing DAPI staining. Furthermore, the influence on ROS production and mitochondrial membrane potential was evaluated at the IC50 concentration using fluorescence microscopy, employing DCFH-DA and Rhodamine 123 dyes.

**Results:**

The synthesis of Au-NPs was confirmed through UV-Vis spectroscopy, with an absorbance peak at 540 nm. FTIR, TEM results showed that the *M. oleifera* mediated Au-NPs had a spherical morphology, and their mean size was approximately 30 nm, as determined by DLS. The Au-NPs exhibited an IC50 value of 50 μg/mL against the A549 lung cancer cells. The DAPI staining results revealed that both concentrations of AuNP, 25 μg/mL and 50 μg/mL, exhibited noteworthy anti-cancer and apoptotic properties.

**Discussion:**

The study demonstrates that M. oleifera-mediated Au-NPs exhibit significant cytotoxic and apoptotic effects on A549 lung cancer cells, with an IC50 value of 50 μg/mL. Both tested concentrations showed substantial anti-cancer properties, as confirmed by DAPI staining. The unique focus on lung cancer, specifically the A549 cell line, sets this study apart from others that address a broader spectrum of cancer types. These findings suggest that M. oleifera-mediated Au-NPs hold promise for clinical applications in lung cancer treatment, providing a potential new therapeutic application.

## Graphical Abstract

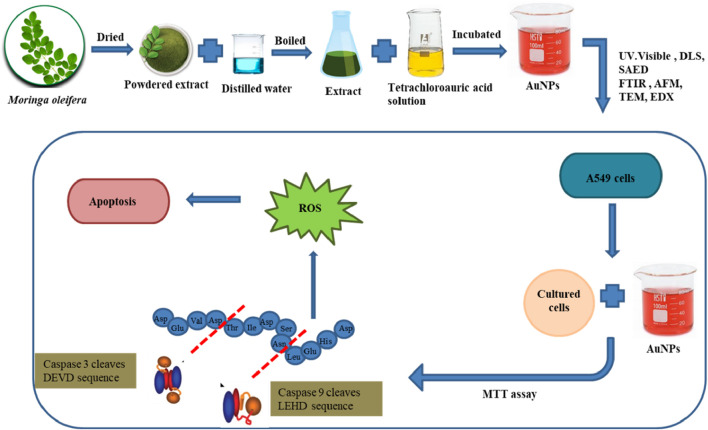

## 1 Introduction

Nanotechnology, a rapidly developing field with a wide range of uses, has greatly broadened its scope to include nanomedicine. The increase in interest can be attributed to the distinctive characteristics of nanoparticles, including their small size at the nanoscale, capacity to be delivered to specific targets, and improved effectiveness (Suganthy et al., 2018). As a result, nanoparticles have become a central focus in medical research and applications. The adaptability and vast utility of nanoparticles are highlighted by their incorporation into many areas such as manufacturing, agriculture, medicine, and everyday products (Maleki et al., 2020; Haddada et al., 2020; Falahati et al., 2020; Liu et al., 2018; Magro and Vianello, 2019; Milincic et al., 2019). Nanoparticles, which include tiny metals such as gold, silver, copper, and titanium, have been widely used in the medical industry (Karuppaiah et al., 2024; Chinnaiah et al., 2022; Karuppaiah et al., 2022; Karthik et al., 2021; Adavallan et al., 2017; Rangayasami et al., 2021).

Au-NPs are widely recognized for their significant applications in the field of medical diagnosis and treatment. Their attractiveness arises from their unique optical properties, chemical durability, compatibility with biological systems, simplicity of production, versatility for functional alteration, and plasmonic traits (Aghaie et al., 2019; Bouche et al., 2020; Ning et al., 2017; Sharifi et al., 2019; Singh et al., 2018b). Gold is generally described as chemically inert and has a high level of biocompatibility in the human body. Nevertheless, its distinctive characteristics are more apparent when its size is reduced to the nanoscale. The transition to nanosized materials results in a significant increase in the surface area-to-mass ratio, improves drug loading capacity and release kinetics. This ensures a more efficient delivery of anticancer drugs to the tumor site (Ahmad et al., 2015). By targeting cancer cells more precisely, nanoparticles can minimize the exposure of healthy tissues to toxic drugs, thereby reducing side effects and improving patient outcomes (Sell et al., 2023; Cheng et al., 2021). The surface plasmon resonance (SPR) phenomenon is the result of the interaction between light and the electrons at the gold surface at this level. The size and shape of the nanostructure are the primary factors that influence the collective oscillation of conduction electrons in response to incident light, which is a defining characteristic of SPR. This phenomenon enables the localised modulation of electromagnetic waves in the vicinity of the material, rendering it highly appropriate for medical applications (Rai et al., 2015). Furthermore, gold nanomaterials are advantageous over other metals due to their modular design approach and the absence of an oxide layer, as well as their extended circulatory circulation. These attributes render gold nanomaterials highly prospective candidates for a diverse array of applications, such as the treatment of lung cancer and other medical conditions (Wu et al., 2018; Zhang et al., 2020; Zeng et al., 2011). The production and integration of Au-NPs has been achieved using various methods, including chemical, physical, and biological methodologies. The biological approach, frequently emphasized for its ecological compatibility, distinguishes itself as a sustainable, minimally harmful, and economically efficient substitute for traditional methods. This technology is renowned for its environmentally friendly attributes, signifying a transition towards more conscientious and ecologically aware approaches in the synthesis of nanomaterials (Khan et al., 2019; Lee et al., 2020; Lopes et al., 2019; Siddiqi and Husen, 2017).

Lung cancer continues to be the most prevalent cause of cancer-related mortality worldwide, resulting in the highest number of fatalities among both men and women. Smoking is the primary risk factor for lung cancer, accounting for approximately 85% of cases. Lung cancer is recognized as the second most prevalent non-contagious ailment, with a prevalence of 50% among men and 30% among women (Rajesh kumar et al., 2018; Wong et al., 2017), underscoring its substantial influence on worldwide health. The significance of early detection and prevention is underscored by the fact that lung cancer is frequently diagnosed at advanced stages, which results in limited treatment options. Primary prevention strategies, including the implementation of tobacco control measures and the reduction of exposure to environmental risk factors, are essential for the prevention of lung cancer and the preservation of life. The International Agency for Research on Cancer (IARC)’s GLOBOCAN 2020 estimates indicate that lung cancer remains the primary cause of cancer-related fatalities, with an estimated 1.8 million deaths worldwide in 2020, which accounts for 18% of all cancer-related mortality (World Health Organization, 2023). Effective preventive and therapeutic interventions are urgently required, as these statistics underscore the substantial public health burden that lung cancer imposes. Conventional therapeutic approaches, including physical therapies, pharmacological interventions, radiation therapy, chemotherapy, and surgical procedures, frequently exhibit limited effectiveness. The emergence of drug resistance in cancer cells is a significant challenge in the successful treatment and control of the disease (Vijayakumar et al., 2019). The development of sophisticated therapeutic alternatives is hindered by the detrimental consequences and toxicity linked to existing medicines, in addition to the financial strain they impose (Anna et al., 2017). The aforementioned concerns highlight the pressing necessity for novel, economically viable, and biologically compatible approaches to address the proliferation of cancer cells, hence facilitating further investigation into potential treatment pathways.

*Moringa oleifera (M. oleifera)*, which belongs to the family Moringaceae, is renowned for its notable velocity of development and extensive geographical range, encompassing regions such as Southwest Asia, Madagascar, Northeast Africa, and Southwest Africa (Abd Rani et al., 2018; Mahmood et al., 2010). This plant is renowned for its abundant nutritional composition and diverse range of beneficial compounds. The results of comparative analysis indicate that *M. oleifera* exhibits notably elevated nutrient concentrations in comparison to commonly consumed foods. Specifically, it possesses 7 times the amount of vitamin C present in oranges, 9 times the protein content found in yoghurt, 10 times the vitamin A content in carrots, 17 times the calcium content in milk, fifteen times the potassium content in bananas, and 25 times the iron content in spinach (Rockwood et al., 2013). *M. oleifera* has shown promising potential in cancer treatments due to its various bioactive compounds (Sultan et al., 2023). *M. oleifera* has been studied for its chemo-preventive properties, which help inhibit the growth of several human cancer cells (Christianto and Smarandache, 2019). Research has indicated that *M. oleifera* can be effective in treating estrogen receptor-positive breast cancer by targeting CDK-2 (Sultan et al., 2023). The plant’s extracts are being explored for their potential to be used in combination with conventional chemotherapy to enhance its efficacy and reduce side effects (Bhadresha et al., 2022). The potential anti-cancer benefits of *M. oleifera* have been revealed through research, particularly by increasing the levels of ROS. ROS activate caspases, p53, and the cleavage of PARP-1, ultimately resulting in apoptosis in different types of malignant cells (Madi et al., 2016). Subsequent investigations have revealed that extracts derived from the leaves of *M. oleifera* has the ability to induce apoptosis in KB carcinoma cells (Sreelatha and Padma, 2011) and impede the proliferation of cancerous cells (Goyal et al., 2007), hence emphasising its potential as a natural therapeutic agent in the context of cancer treatment.

Projections for 2027 suggest a 70% rise in cancer mortality, primarily caused by lung, breast, prostate, and colorectal cancers. Lung cancer, in isolation, accounts for 25% of all cancer-related fatalities, exhibiting a significantly low survival rate of merely 17.8% (National Cancer Institute, 2022). According to the National Cancer Center (NCC), China experiences an annual occurrence of around 781,000 instances of lung cancer (National Cancer Center, 2019). Chemotherapy and radiotherapy have long been recognized as the primary therapeutic approaches for lung cancer (Curran et al., 2011), effectively improving patient survival rates. However, their effectiveness is hindered by notable adverse effects. This is mostly due to the fact that these therapies do not solely focus on malignant cells (Li et al., 2018), also impact healthy cells. A significant obstacle associated with conventional allopathic medicine is to the metabolism and medication clearance rate, which might impose constraints on the efficacy of therapeutic interventions (Sinha et al., 2006). Nanoparticles present a potentially viable resolution to these constraints, since they possess the ability to selectively target cancer cells with more accuracy, hence potentially mitigating the adverse effects commonly associated with conventional chemotherapy and radiotherapy. The main purpose of this study is to study the efficacy of nanopartized Au-NPs derived from *M. oleifera* as a novel therapeutic approach for lung cancer *in vitro*.

## 2 Materials and methods

### 2.1 Chemicals

For the experiment, the following materials were obtained from Sigma-Aldrich, China: tetrachloroauric (III) acid, fetal bovine serum, RPMI 1640 cell culture medium, trypsin, thiazolyl blue tetrazolium bromide (MTT), and penicillin-streptomycin solution.

### 2.2 Preparation of plant extract

The *M. oleifera* leaves, obtained from a local farm and cleaned thoroughly using distilled water and were thereafter allowed to air-dry in a shady location for a duration of 15 days until complete desiccation was achieved. Following this, the dried leaves were pulverized into a fine powder using an electric mixer. A mass of 100 g of the powdered *M. oleifera* was thereafter subjected to boiling in 1 L of distilled water at 60°C for 30 min. After undergoing this procedure, the resulting extract was subjected to filtration and thereafter set aside for further analysis and utilization.

### 2.3 Synthesis of Au-NPs

The synthesis of Au-NPs was carried out by combining 10 mL of an aqueous extract of *M. oleifera* with 190 mL of a 1 mM HAuCl4 solution. The reaction mixture was maintained at ambient temperature for 15 min without any disturbance. The UV-vis absorption spectrum was used to monitor the reduction of AuCl_4_^−^ ions over time. The solution’s colour changed from yellow to deep ruby red, indicating the successful formation of Au-NPs (Sindhu et al., 2014). The nanoparticles were centrifuged at 14,000 rpm for 20 min at room temperature after the reaction was finished to remove large aggregates. The supernatant was collected, purified using PD-10 columns (GE Healthcare, Chicago, IL, United States), and fractionated into 3.5 mL eluates. Subsequently, these samples were dialyzed against a 10 mM sodium phosphate buffer (pH 7.0) using 20 kDa molecular weight cutoff dialysis bags. The dialysis procedure consisted of a buffer exchange after 2 h, which was followed by an incubation period of 15–18 h.

### 2.4 Characterisation

After the synthesis and purification process, the Au-NPs were subjected to a series of analytical tests to confirm their dimensions, morphology, and structural characteristics. The validation of the Au-NPs was conducted using Ultraviolet-visible (UV-Vis) spectroscopy. A UV-1800 Shimadzu spectrophotometer was used to measure the absorbance within the wavelength range of 300–700 nm. The utilization of transmission electron microscopy (TEM JEOL JEM-1230) yielded precise visual representation, validating the measurements and morphology of the Au-NPs. The stability and crystallinity of the nanoparticles were evaluated using energy-dispersive X-ray analysis (EDX), while the surface characteristics and overall size distribution of the nanoparticles were assessed by selected area electron diffraction (SAED) using Zetasizer Pro (Malvern P analytical Ltd., UK). Dynamic Light Scattering and Zeta Potential using Zeta-PAL (Brookhaven, USA) were performed to know the size and surface charge of prepared Au-NPs. The functional groups present in the Au-NPs were identified using FTIR instrument Invenvio (Bruker). The FTIR spectral data was acquired between 4,000 and 1,000 cm^−1^ using the KBr pellet method. The extensive range of analysis conducted in this study offered a full evaluation of the Au-NPs, including several aspects such as their physical, chemical, and structural properties. The powdered XRD analysis of synthesized Au-NPs was performed by using Bruker AXS X-ray diffractometer with diffraction intensities at 2ϴ range from 10°–80°

### 2.5 Anti-cancer studies

#### 2.5.1 Culturing of A549 lung cancer cells

A549 cells, which are derived from human adenocarcinomic alveolar basal epithelial cells, have been crucial in advancing the study of lung cancer biology, particularly in the areas of tumor progression, metastasis, and therapeutic response. These cells are frequently used because of their consistent and reproducible experimental results, as well as their simplicity of cultivation in laboratory setting (Giannopoulos-Dimitriou et al., 2024). The A549 cell line used in the present study was obtained from the Institute of Biochemistry and Cell Biology, Chinese Academy of Sciences, Shanghai, China. Throughout the duration of the study, the cells were cultured in Dulbecco’s Modified Eagle Medium (DMEM)/F12. The culture media was supplemented with 10% fetal bovine serum and 1% antibiotic-antimycotic solution (consisting of penicillin and streptomycin) to promote cellular proliferation and protect against microbial pathogens. The A549 cells were cultivated in a CO_2_ incubator maintained at a temperature of 37°C and immersed in a 5% CO_2_ environment. The medium underwent refreshment at intervals of 48 h or earlier in the event of a yellow discoloration, which signifies a change in pH or depletion of nutrients. Once the cell density reached around 80% confluence, which signifies a culture that is healthy and actively growing, the cells were ready for passaging. To allow their transfer to other culture vessels for further growth, the cells were detachable using a 0.25% trypsin solution with 0.02% ethylenediaminetetraacetic acid (EDTA).

#### 2.5.2 MTT assay

A dose-response study was conducted to evaluate the cytotoxicity of Au-NPs on A549 lung cancer cells. The study employed doses ranging from 1 to 50 μg/mL and subsequently conducted an MTT assay (Mosmann, 1983). To assess the influence of Au-NPs on mitochondrial integrity, the production of formazan crystals was observed. These crystals are formed as a consequence of the reduction of MTT by mitochondrial succinate dehydrogenase, which is commonly released by hepatic cells. In this experimental study, cells were dissociated using trypsin and subsequently measured using a cell counter. The aim was to allocate roughly 10^5^ cells per well in 96-well plates. The lung cancer cells were exposed to various concentrations of Au-NPs, ranging from 0 to 50 μg/mL, and then placed in a 37°C environment with a 5% CO_2_ atmosphere for a duration of 24 h. The determination of cell survival rates was conducted using the MTT test, which allowed for the identification of the median cytotoxic concentration (IC50) of Au-NPs for further inquiry.

#### 2.5.3 DAPI staining

The investigation into the anti-cancer properties of biosynthesized Au-NPs entailed an analysis of the alterations in cell shape following exposure to Au-NPs. The TUN staining was used to accomplish this. Each well of 6-well plates was seeded with approximately 200,000 cells, which were subsequently treated to doses of 15 and 20 μg/mL of Au-NPs. After being incubated in a 5% CO_2_ atmosphere for 24 h at 37°C, the cells were fixed for 15 min using a solution containing 2.5% glutaraldehyde and 0.1% Triton X-100. Following fixation, the cells were subjected to DAPI staining for 5 min at 37°C. This process aimed to emphasise the nuclei, making it easier to detect any alterations that may indicate apoptosis or necrosis. The cells were rinsed with ice-cold PBS after DAPI staining in order to eliminate any remaining stain. The NIKON Eclipse 80i fluorescent microscope was utilised to conduct observations on the labelled lung cancer cells, facilitating a comprehensive visualisation of the cellular reactions to Au-NPs treatment.

#### 2.5.4 Caspases activity

Assays were conducted utilising kits obtained from China to assess the effects of Au-NP on caspases, which are crucial proteins that trigger apoptosis. The experiments employed the p-NA substrate, which emits light when broken down by caspases. The DEVD sequence is recognised and cleaved by caspase 3, whereas the LEHD sequence present in the p-NA substrate is targeted and cleaved by caspase 9. This process leads to the generation of light, which was measured at a wavelength of 400 nm. This technique enables the quantification of caspase activity, therefore evaluating the inducer of cell death caused by Au-NPs.

#### 2.5.5 ROS damage

The fluorescent probe known as 2′,7′-Dichlorodihydrofluorescein diacetate (H_2_DCFDA) is a derivative of fluorescein that exhibits cellular permeability. It functions as a highly sensitive detector for ROS. The conversion of H_2_DCFDA from a non-fluorescent state to the highly fluorescent 2′,7′-dichlorofluorescein (DCF) occurs upon its contact with ROS. This transformation enables the quantification of H_2_DCFDA at a wavelength of 530 nm (Klastersky et al., 1989). The conversion of ROS is of utmost importance in the investigation of the impact of anti-cancer drugs on the apoptotic process. The presence of Au-NPs has been demonstrated to increase levels of ROS, which can be accurately measured using the H2DCFDA assay kit. The experiment involved cultivating A549 cells in 6-well plates and subjecting them to Au-NPs at dosages of 15 and 20 μg/mL for a period of 24 h. After the treatment, the cells were incubated at 37°C for 30 min with a concentration of 80 mM of the H_2_DCFDA dye. Following that, the fluorescence intensity at 530 nm was measured using an ELISA reader. The results for each sample were corrected according to the protein content in order to maintain accuracy.

#### 2.5.6 Mitochondrial damage

The influence of Au-NPs therapy on mitochondrial membrane integrity in A549 cells was disclosed using fluorescence microscopy, with rhodamine 123 serving as a selective marker (Rajivgandhi et al., 2018b). The observation of cyt-c efflux from the mitochondria into the cytosol upon exposure to the IC50 concentration of Au-NPs is a significant milestone in the process of apoptosis, indicating the potential anti-cancer properties of Au-NPs. This phase highlights the initial interaction between Au-NPs and A549 cells, resulting in the disruption of mitochondria. The cell cultures that had reached maturity on coverslips were subjected to the IC50 concentration of Au-NPs and thereafter incubated for 24 h at 37°C. After the incubation period, the cells were subjected to centrifugation and subsequently subjected to trypsinization. Following the process of trypsinization, a washing step was conducted using 1x PBS. The cells on coverslips were treated with Rhodamine 123 dye at a dosage of 5 μg/mL for a duration of 1 h. Following this time frame, any excess dye was eliminated using Whatman No.1 filter paper and 1x PBS. In order to differentiate between the modified structures of impaired and undamaged mitochondrial membranes in the A549 cell line, a fluorescent microscope was employed to investigate both the treated and control cell groups.

### 2.6 Statistical analysis

The data were analysed using one-way ANOVA to determine statistical significance between groups, and all experiments were conducted in triplicate. The GraphPad Prism software was employed to conduct the analysis. Statistical significance is established at p < 0.05, and the results are presented as the mean ± standard deviation (SD).

## 3 Results and discussion

### 3.1 Characterisation

#### 3.1.1 UV-visible spectroscopy

**FIGURE 1:**
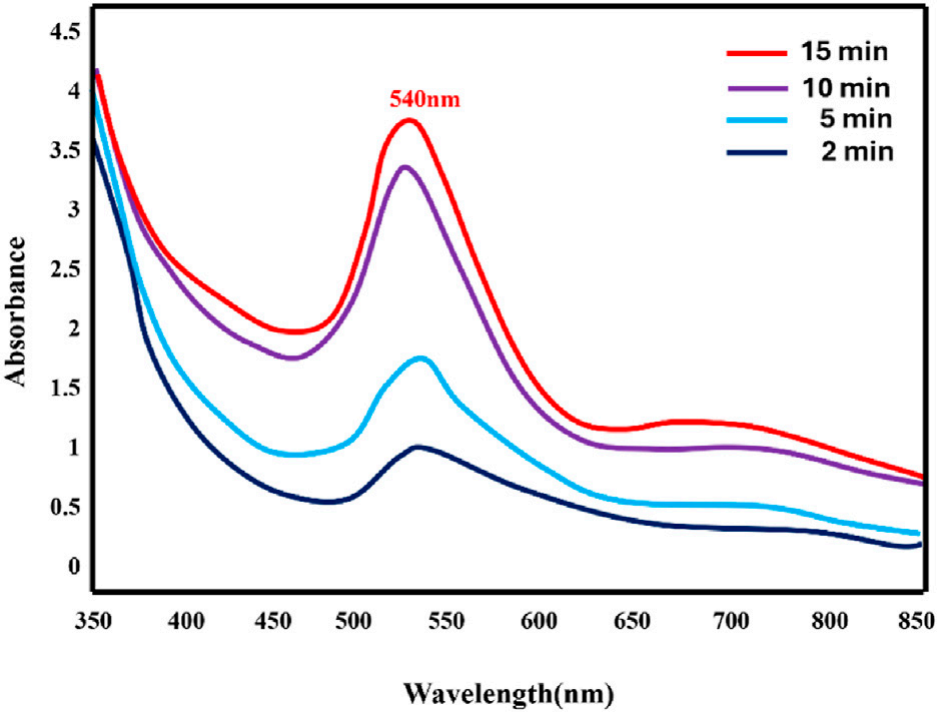
UV spectra of Au-NPs synthesised using *M. oleifera*.

The synthesis of Au-NPs was confirmed using UV-Vis spectroscopy. The absorbance spectra exhibited a range of values between 350 and 700 nm, with the most prominent peak observed at 540 nm as depicted in Figure 1. It is found that the Au-NPs formation was completed within 15 min at room temperature. However, when the reaction temperature was maintained at 100°C, the reaction was completed within 5 min (Supplementary Figure S1). This peak is a direct indication of the SPR effect, confirming the formation of Au-NPs. This peak is within the expected range for gold nanoparticles, indicating successful synthesis. The size and shape of the nanoparticles were exhibited depending upon the SPR property of the metal (Fayaz et al., 2010).

#### 3.1.2 Fourier transformed infrared spectroscopy

**FIGURE 2:**
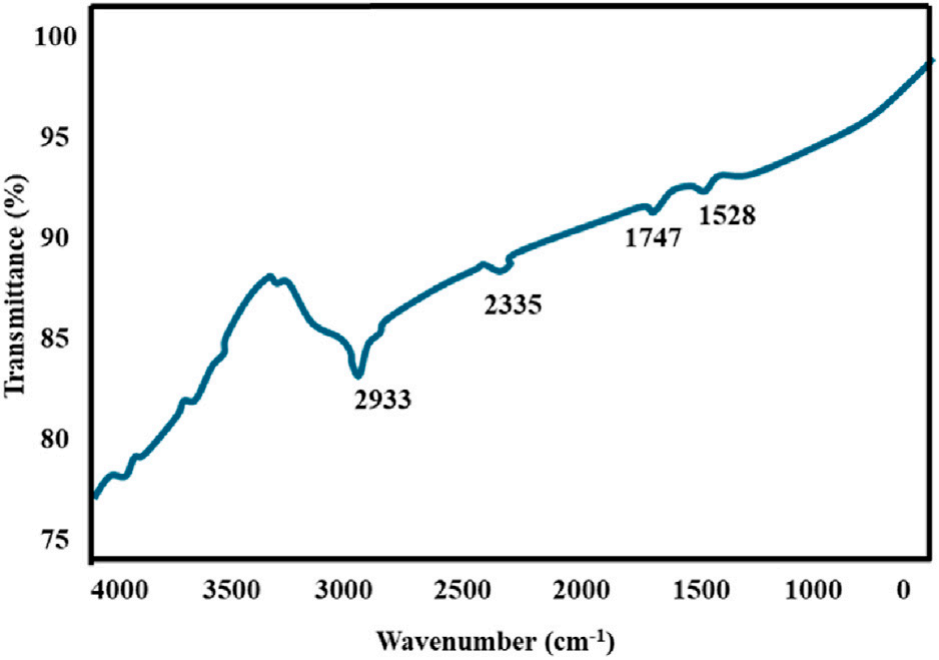
FTIR spectra of Au-NPs bio fabricated using Aqueous leaf extract of *M. oleifera*.

The biomolecules included in the Au-NPs samples were identified and their ability to create a capping layer was assessed using FTIR spectroscopy (Figure 2). Prominent absorption peaks were seen in the spectrum analysis, notably at 1,528, 1,747, 2,335, and 2,933 cm^−1^, within the range of 1,000–4,000 cm^−1^. The observed peaks are caused by the interaction and reduction of gold ions due to the presence of compounds in *M. oleifera*. The presence of hydroxyl groups in polyphenolic compounds from *M. oleifera* is indicated by the absorption peak at 2,933 cm^−1^. The peak observed was due to the decrease of gold content which may be due to the presence of polyphenols that binds the gold with carbonyl group and cap the Au-NPs hence preventing from combination of nanoparticles (Ali et al., 2011). The functional components of *M. oleifera* could have bound to AuCl_4_ and decreased it to the formation of Au-NPs. Additionally, the inclusion of ketones, aldehydes, and esters within the nanoparticle structure is suggested by the peak at 2,335 cm^−1^. Polyphenols, including quercetin and gallic acid, have been shown to inhibit cancer cell growth and induce apoptosis in lung cancer cells (Mumtaz et al., 2021). Flavonoids like kaempferol and catechins have demonstrated anti-inflammatory and anti-proliferative effects on lung cancer cells. They can modulate signalling pathways involved in cancer cell survival and proliferation (Wu et al., 2021). Phenolic compounds can enhance the immune response against cancer cells by promoting the activity of immune cells such as T-cells and macrophages. They also help in reducing tumor-associated inflammation (Wang et al., 2023). Isothiocyanate**s** found in *M. oleifera*, have been shown to inhibit cancer cell migration and invasion, thereby preventing metastasis (Wu et al., 2021).

#### 3.1.3 Transmission electron microscopy

**FIGURE 3:**
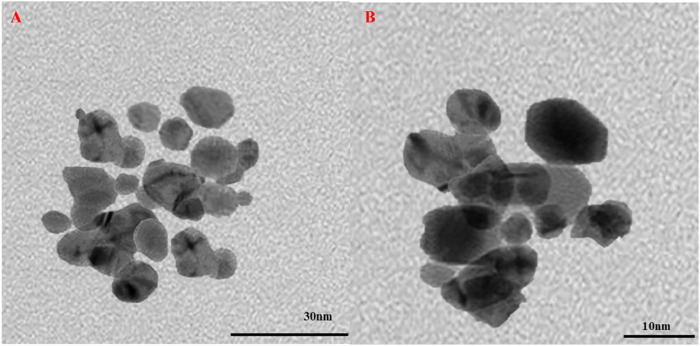
TEM images of bio synthesised Au-NPs using *M. oleifera* **(A)** 30 nm **(B)** 10 nm.

TEM was used to assess the shape and structural characteristics of Au-NPs obtained from the leaf extract of *M. oleifera*. Figures 3A, B presents TEM images illustrating Au-NPs with diameters varying between 20 and 40 nm, most of them displaying a spherical shape. The spherical morphology of Au-NPs observed in the TEM results provides several advantages for biomedical applications, including enhanced stability, dispersibility, cellular uptake, and functionalization capabilities (Georgeous et al., 2024). These properties make spherical Au-NPs particularly suitable for targeted drug delivery and cancer therapy.

#### 3.1.4 Energy dispersive X-ray analysis

**FIGURE 4:**
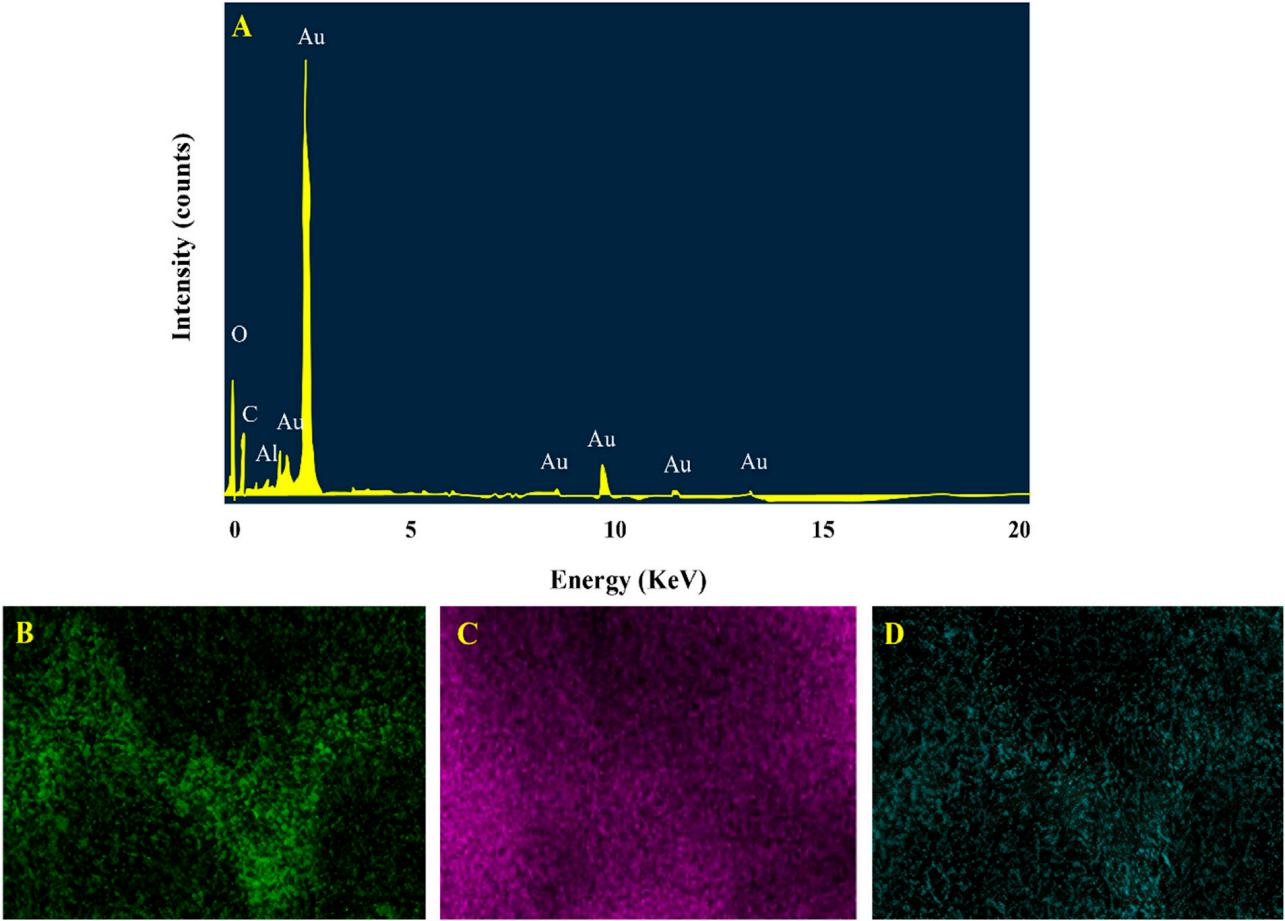
EDX pattern of Au-NPs **(A)** Elemental mapping of gold nanoparticles with corresponding elements such as Gold **(B)** Oxygen **(C)** Carbon **(D)**.

The production and stability of Au-NPs by plant-derived phytochemicals were further confirmed using EDX examination. The EDX profile of Au-NPs as depicted in Figure 4 exhibited strong Au signals corresponds to elemental Au-NPs and additional peaks corresponding to carbon, oxygen were also observed. These peaks may be attributed to biomolecules present in the extract (Moodley et al., 2018). The elemental percentages were shown in Table 1.

**TABLE 1:** EDX elemental composition of AuNPs.

Element	Line	Mass%	Atom%
C	K	27.18	64.50
O	K	14.53	25.95
Al	K	1.20	1.30
Au	M	57.09	8.25
Total		100.00	100.00

#### 3.1.5 Selected area electron diffraction

**FIGURE 5:**
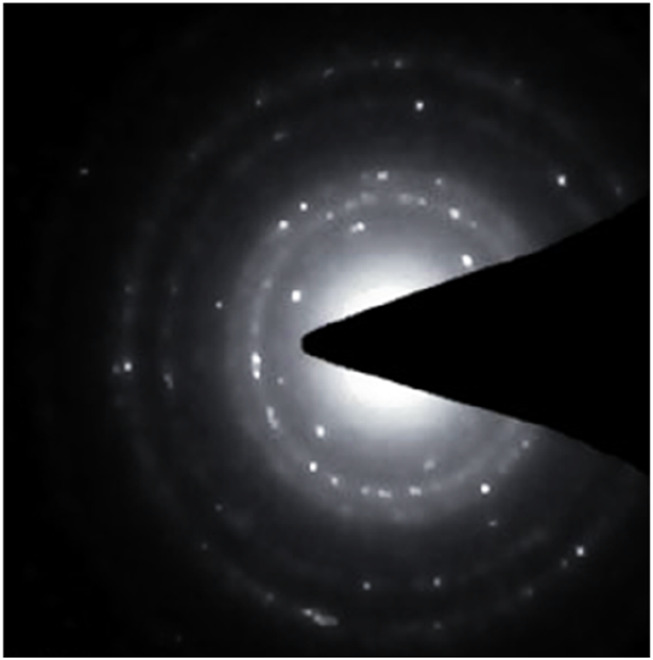
SAED pattern of biosynthesised Au-NPs bio synthesised using *M. oleifera*.

The study further confirmed the crystallinity of the Au-NPs using (SAED) analysis, as shown in Figure 5. The pattern seen in the SAED analysis exhibited prominent, concentric rings, which provide as evidence for the crystalline arrangement of the Au-NPs. The diffraction patterns observed in these rings are a result of the different lattice planes present in the crystalline Au-NPs. SAED pattern is attributed to the (111), (200), (220) and (311) planes of face centered cubic compatible with the XRD.

#### 3.1.6 X-ray diffraction technique

**FIGURE 6:**
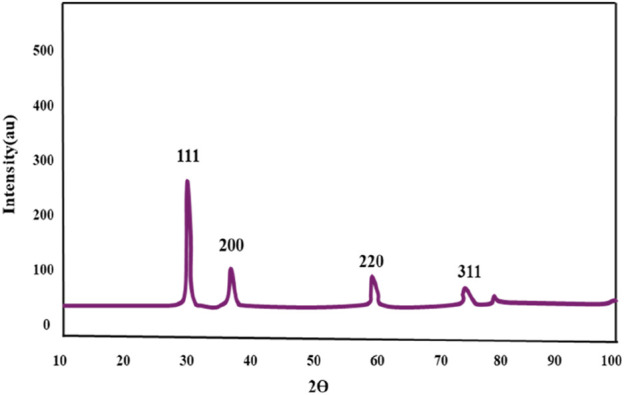
XRD pattern of AuNPs bio synthesised using *M. oleifera*.

The crystalline structure of the Au-NPs was definitively determined using XRD research. The XRD spectrum, as illustrated in Figure 6, exhibited four distinct diffraction peaks at angles of 38.12°, 44.87°, 64.77°, and 77.23°. The peaks are indicative of the lattice plane indices (111), (200), (220), and (311), which are indicative of the face-centered cubic (FCC) structure of gold (Au). The measured lattice parameter of a = 4.08A° exhibits a strong agreement with the established values for FCC gold, as supported by the reference data obtained from JCPDS Card No-04-0784.

#### 3.1.7 Dynamic Light Scattering (DLS) analysis

**FIGURE 7:**
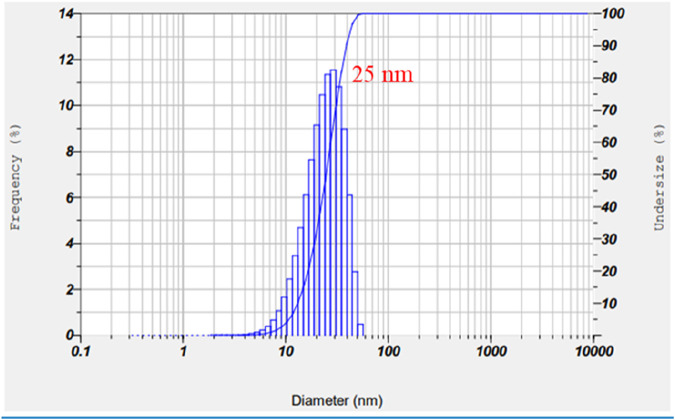
DLS graph of biosynthesised Au-NPs bio synthesised using *M. oleifera*.

DLS analysis was used to determine the overall size and thickness of the capping layer on the nanoparticles. The mean size of the Au-NP was determined to be around 30 nm using this methodology, as depicted in Figure 7. The polydispersity index, which quantifies the range of particle sizes, was found to be approximately 0.321%. The results of the characteristic analysis were compared with the results of the previous studies, showed in Table 2 (Guliani et al., 2021; Khan et al., 2021; Gopinath et al., 2014).

**TABLE 2:** Comparison table of characterization results with previous studies.

Name of the plant extract	Uv-visible results	FTIR- results	TEM results	DLS results	EDX results	Reference
*Populus alba*	∼540 nm	3304 (O-H), 1628 (C=C)	Spherical	20–30 nm	Au, C, O	Guliani et al. (2021)
*Lantana camara*	530–570	3400 (O-H), 1600 (C=C)	Spherical	10–20 nm	Au, C, O	Guliani et al. (2021)
*Erythrophyleum guineense*	530–570	3400 (O-H), 1600 (C=C)	Irregular round	<100 nm	Au, C, O	Khan et al. (2021)
*Hibiscus arboreus*	530–570	3400 (O-H), 1600 (C=C)	Spherical	30–40 nm	Au, C, O	Guliani et al. (2021)
*Terminalia arjuna*	523 nm	3400 (O-H), 1600 (C=C)	Spherical	20–50 nm	Au, C, O	Gopinath et al. (2014)
*Delphinium uncinatum*	530–570	3400 (O-H), 1600 (C=C)	Irregular round	<100	Au, C, O	Khan et al. (2021)

### 3.2 Anti-cancer studies

#### 3.2.1 IC50 dose analysis

**FIGURE 8:**
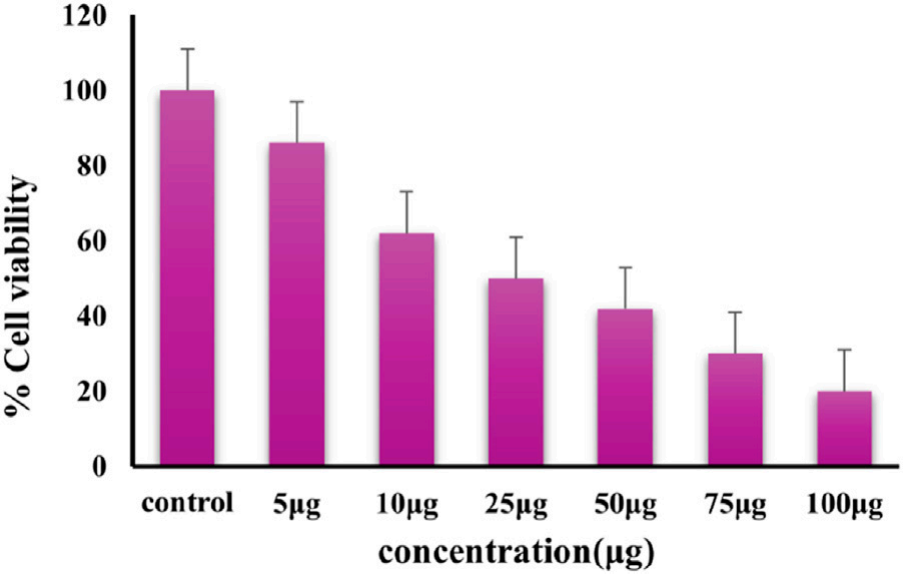
Cytotoxicity effect of Au-NPs synthesized using *M. oleifera*.

The cytotoxic effects of different doses of Au-NPs on the lung cancer A549 cell line are depicted in Figure 8, which was evaluated using the MTT assay. Determining the IC50 value helps in identifying the optimal dose range for a drug candidate. Using IC50 values helps in finding the lowest effective dose that minimizes harmful effects on normal cells (IC50, 2025; Robles-Bañuelos et al., 2024). IC50 value was obtained at dose of 50 μg/mL which inhibited the growth of 50% A549; hence, this dosage was selected for the analysis of anticancer effect of Au-NPs. In order to further investigate the impact of Au-NPs on cell survival, a concentration of 50 μg/mL was used for following studies, as well as a reduced concentration of 25 μg/mL. The high concentration of bioactive metabolites in *M. oleifera* leaf extracts, which substantially contribute to the induction of cytotoxicity, can be attributed to the cytotoxic effect of Au-NPs (Gismondi et al., 2015). Furthermore, the interaction between gold atoms and intracellular components plays a crucial role. Gold ions disrupt cellular function and contribute to the observed cytotoxic effects by interfering with intracellular proteins, as well as phosphate and nitrogen groups in DNA (Blagoi et al., 1991). In the previous studies phenolic compounds of *M. oleifera* leaf extracts were tested for their anti-cancer activity against Hela cancer cells in which n-hexane fraction showed a 50% reduction in Hela cancer cell viability at 416 μg/mL compared to control (Mumtaz et al., 2021). Some previous studies reported an IC50 value of 11.13 μg/mL for AS1411 aptamer-conjugated, green-synthesized AU-NPs against MCF-7 breast cancer cells (Helal et al., 2024) and 10 μg/mL for Au-NPs combined with metformin against MCF-7 and A549 cells (Yeşildağ et al., 2024).

#### 3.2.2 Apoptic effect of Au-NPs

**FIGURE 9:**
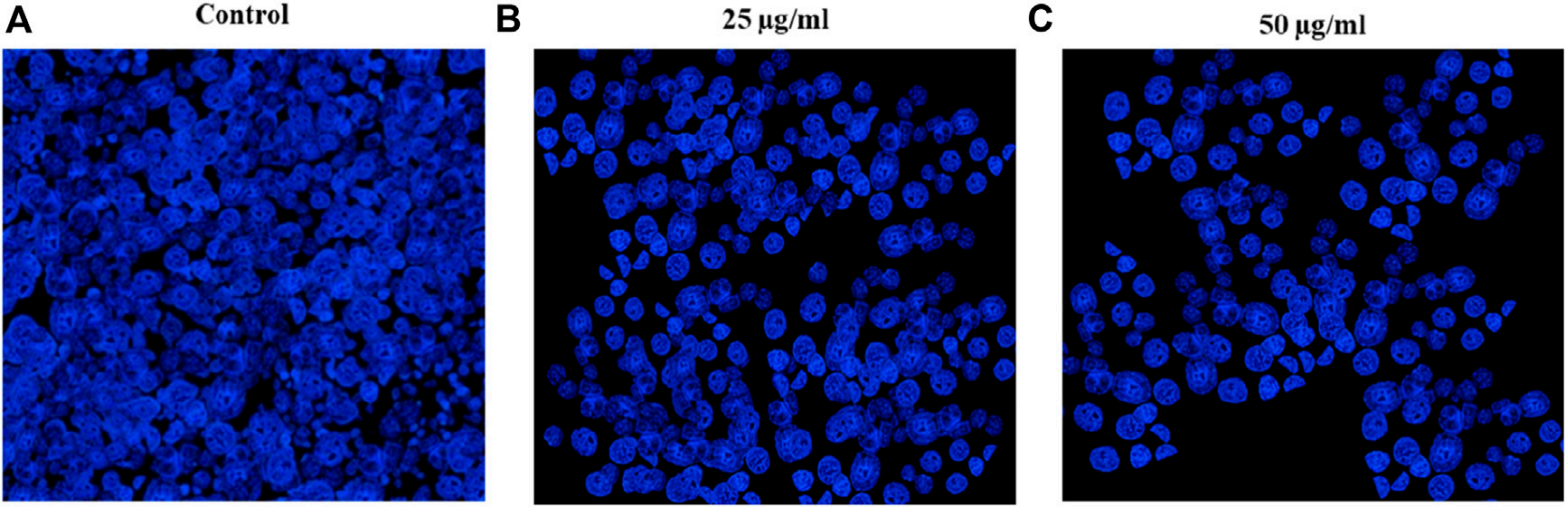
Apoptotic changes induced by Au-NPs synthesized using *M. oleifera* evidenced with DAPI staining **(A)** control **(B)** 25 μg/mL **(C)** 50 μg/mL.

The study examined the ability of Au-NPs to induce apoptosis in the lung cancer A549 cell line using DAPI labelling. The DAPI staining findings are depicted in Figures 9A–C, illustrating the comparison between control cells and cells treated with Au-NPs. The nuclei in the control samples were found to be undamaged, whereas the application of Au-NPs at doses of 25 μg/mL and 50 μg/mL led to a decrease in the cell viability. Au-NPs can generate ROS which are oxygen-containing molecules with a high degree of chemical reactivity. Oxidative stress can be induced by the accumulation of ROS, which can result in the destruction of critical cellular components, including DNA, proteins, and lipids (Foglietta et al., 2023). The release of pro-apoptotic factors, such as cytochrome c, into the cytoplasm can be a consequence of elevated ROS levels, which can impair mitochondrial function. The activation of caspases, a class of proteases that mediate apoptosis by cleaving specific cellular targets, is initiated by this event (Lee et al., 2022). Furthermore, ROS have the potential to cause DNA damage, which in turn activates the p53 pathway, thereby contributing to apoptosis and cell cycle arrest (Foglietta et al., 2023; Lee et al., 2022). Elevated ROS levels, caspase activation, DNA and protein degradation, and alterations in the cell membrane are among the primary biochemical changes associated with apoptosis (Kumar et al., 2010). An increase in intracellular ROS levels and activities of caspases 3 and 9 has been observed in lung cancer cells treated with Au-NPs, confirming the induction of apoptosis in the A549 lung carcinoma cell line. This apoptotic process was further verified by DAPI staining. Previous studies revealed that Au-NPs induced apoptosis and inhibited cancer cell proliferation (Siddique et al., 2022).

#### 3.2.3 Au-NPs effect on caspases activity

**FIGURE 10:**
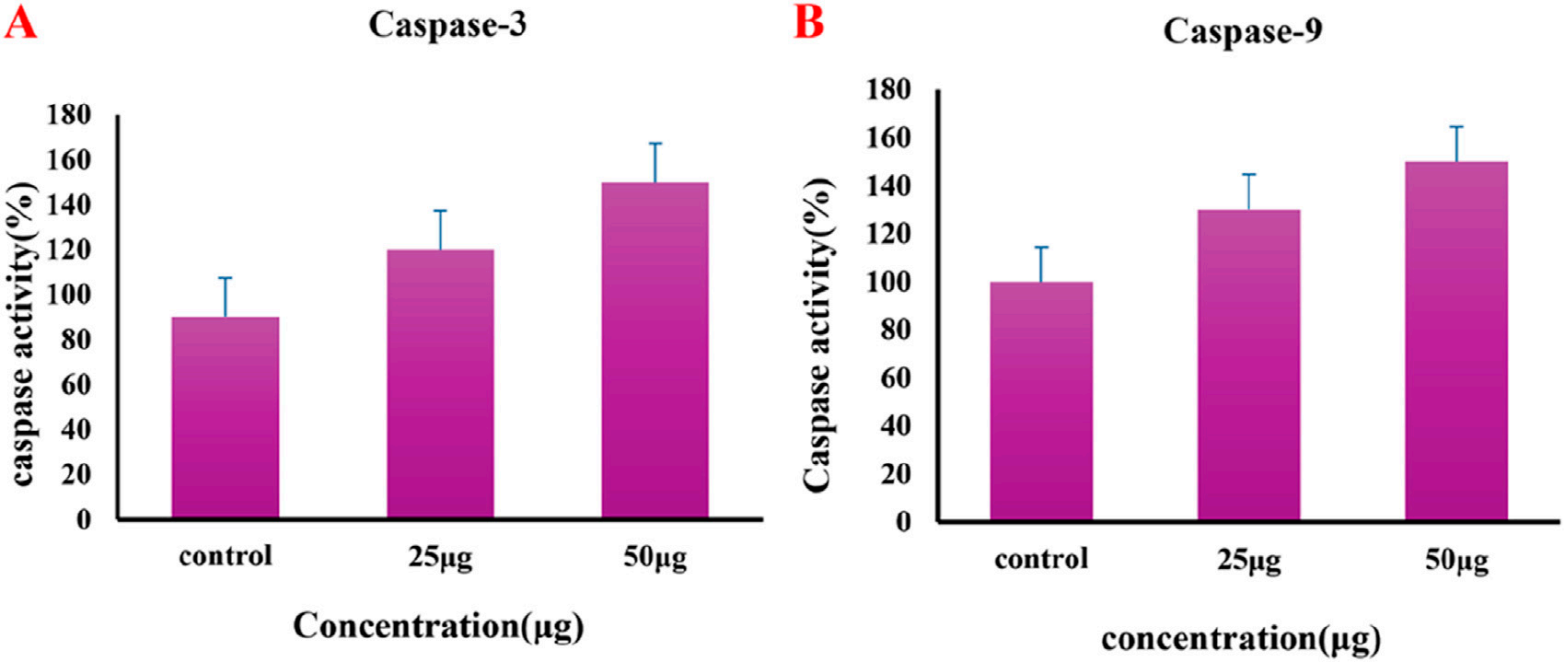
Effect of Au-NPs biosynthesized using *M. oleifera* on induction of caspases **(A)** Caspase-3 **(B)** Caspase-9.

In the process of apoptosis, caspases play a vital role, wherein Caspase 3 acts as an initiating caspase and Caspase 9 acts as an executing caspase. The caspase activity in untreated control cells and lung cancer A549 cells subjected to different dosages of Au-NPs is depicted in Figures 10A, B. A549 cells exposed to concentrations of Au-NP at 25 and 50 μg/mL exhibited a notable elevation in both initiating and executing caspase levels. Specifically, cells treated with 25 μg/mL of Au-NPs shown a nearly 1.5-fold increase in caspase activity. The data presented provide evidence of the significant apoptotic impact of Au-NPs on lung cancer cells through the augmentation of caspase activation. The anticancer activity of Au-NPs is strongly supported by the elevated levels of caspase 3 in A549 cells. An increase in the protein expression of Beclin 1, a mammalian orthologue of yeast Atg6, further corroborated this effect. Beclin 1 is essential for the regulation of apoptosis, and its activity is inhibited by Bcl2, which binds to the BH3 domain of Beclin 1 to suppress its function (Kang et al., 2011). Treatment with AuNPs led to a decrease in Bcl2 protein expression, which likely facilitated the elevated levels of Beclin 1, thereby inducing apoptosis in the A549 lung carcinoma cell line.

#### 3.2.4 ROS generation

**FIGURE 11:**
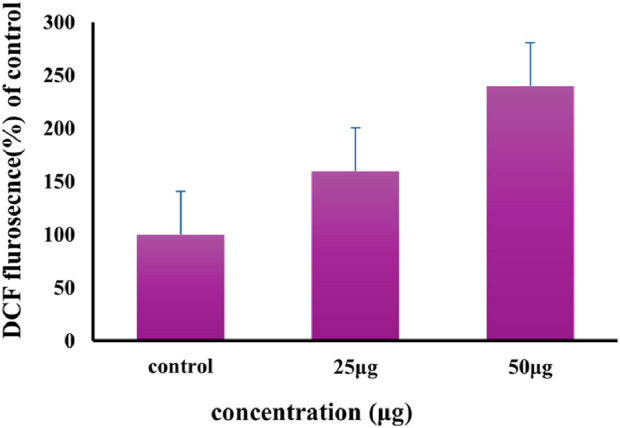
Effect of Au-NPs biosynthesized using *M. oleifera* on ROS production in A549 lung cancer cell line.

This study illustrates the comparison of ROS levels in A549 lung cancer cells that were treated with Au-NPs and those that were not treated (control). The administration of Au-NPs at concentrations of 25 μg/mL and 50 μg/mL led to a 50% and 100% rise in ROS generation, indicating that the biosynthesized Au-NPs induces apoptosis (Figure 11). The observed rise in ROS levels is associated with an elevated occurrence of apoptotic cells as identified by DAPI staining. Activators of the intrinsic apoptotic pathway are recognised for their ability to elevate intracellular ROS and cytosolic calcium levels (Kang et al., 2011). The levels of ROS in A549 cells were substantially increased by the treatment with Au-NPs in this study. The protein expression of intrinsic apoptotic signalling molecules was analysed in both control and Au-NPs treated cells to further investigate the mechanism. A significant change was observed in the Bcl2 family of proteins, which encompasses both pro-apoptotic and anti-apoptotic members. The pro-apoptotic proteins Bid and Bax were upregulated, while the anti-apoptotic protein Bcl2 was downregulated (Karp, 2008). It is well-established that Au-NPs catalyse the production of ROS through electron transfer reactions, which leads to the formation of reactive species including superoxide anions, hydrogen peroxide, and hydroxyl radicals. AuNPs can also discharge metal ions that contribute to the generation of ROS. The cellular antioxidant defences can be overpowered by the elevated ROS levels, which can result in oxidative stress and cellular injury (Reed, 1997). The importance of ROS in the targeting of cancer cells has been emphasised in previous research. For example, Naveen kumar et al. (2018b) demonstrated that AgNPs significantly induced oxidative stress responses in A549 cells, thereby establishing ROS as a critical factor in cancer inhibition. In the same vein, Bhakya et al. (2016) reported that the integrity of the mitochondrial membrane was disrupted in A549 cells as a result of the oxidative stress induced by higher concentrations of AgNPs. This resulted in the downregulation of anti-apoptotic genes, which in turn triggered programmed cell death through mitochondrial dysfunction and intracellular leakage.

#### 3.2.5 Mitochondrial damage

After 24 h of incubation period, the use of fluorescence microscopy demonstrated the presence of cyt-c leakage from the mitochondrial membrane subsequent to the application of Rhodamine 123 labeling, as depicted in Supplementary Figures S2A, B. Rhodamine 123 has been acknowledged for its effectiveness in detecting changes in the membrane structure of malignant cells through the disruption of the intrinsic route of the cancer cell cycle. This dye exhibits specific affinity for dysfunctional mitochondrial membranes, hence identifying the impacted cells. Cellular damage results in the development of apoptotic and necrotic cells, which exhibit a noticeable change in fluorescence from orange to green. This change emphasizes the important observation of mitochondrial membrane depolarization caused by gene failure. The activation of inhibitory genes, including as BCL-2 and caspases, is initiated by damage to the mitochondrial membrane, resulting in a reduction in gene production that facilitate the proliferation of cancer cells. Introduction of Rhodamine 123 into the mitochondrial membrane leads to the removal of internal leakage materials and a color transition from red to green, which signifies apoptosis (Momtazi-borojeni et al., 2013). The findings of this investigation provide additional evidence of the inhibition of A549 cell proliferation at the IC50 concentration of Au-NPs. The treated samples exhibited a significant rise in apoptotic and necrotic cells when compared to the untreated control group, as depicted in Supplementary Figures S2A, B. This finding suggests that the apoptotic cell receptor cascade is activated in A549 cells, leading to a notable disruption in the course of the cell cycle. This disruption is attributed to an increased activation of gene response, which signifies the onset of apoptosis. The results presented in Supplementary Figure S2B illustrate the presence of rough, loosely connected apoptotic cells exhibiting a clear necrotic feature, while Supplementary Figure S2A showcases smooth, densely packed cell colonies. These findings align with the results reported by Rajivgandhi et al. (2019b) in which observed reduction in red fluorescence intensity indicates a notable decline in the integrity of the mitochondrial membrane. The efficacy of cancer cell therapy procedures is attributed to the loss and functional degradation of the mitochondrial membrane, which results in heightened cell mortality. The findings of our study are consistent with the research conducted by Du et al. (2017), which highlights the significance of mitochondria in initiating cellular signaling pathways, promoting cell differentiation, inducing apoptosis, and regulating the course of the cell cycle. In Table 3 we have provided the comparison table of previous studies (Liu et al., 2013; Niloy et al., 2021; Ramalingam et al., 2017; Tanino et al., 2020; Rani et al., 2022; Hongal et al., 2024; Dehnoee et al., 2024; Kalaiarasi et al., 2018).

**TABLE 3:** Comparison table of results with previous studies.

Nanoparticle	Mechanism	Results	References
AuNPs	Apoptosis, necrosis	Induced apoptosis and necrosis	Bhakya et al. (2016)
AuNPs	Endocytosis, imaging, drug delivery	Improved diagnostic accuracy, targeted therapy	Momtazi-borojeni et al. (2013)
AuNPs	Inducing apoptosis, cell cycle arrest	Significant apoptosis and cell cycle arrest	Rajivgandhi et al. (2019b)
ZnO NPs	Genotoxicity, ROS generation	Low viability, high ROS generation	Du et al. (2017)
ZnO NPs	Apoptosis, cell cycle arrest	High apoptosis, G1-phase arrest	Liu et al. (2013)
AgNPs	Inducing apoptosis, cell cycle arrest	Significant apoptosis and cell cycle arrest	Niloy et al. (2021)
CuONPs	Growth inhibition, cytotoxicity	Significant growth inhibition, low cytotoxicity	Ramalingam et al. (2017)
CuONPs	Cell cycle arrest, DNA damage	Induced cell cycle arrest, DNA damage	Tanino et al. (2020)

While the present findings provide a promising foundation for lung cancer treatment, further studies are necessary to evaluate the *in vivo* safety, biocompatibility, and potential toxicity of these nanoparticles. Addressing these concerns is critical for the translation of these results into clinical applications.

## 4 Conclusion

In conclusion, biocompatible and environmentally benign methods were employed to produce Au-NPs by utilising extracts from the *M. oleifera* plant. A visible colour transformation of the solution to ruby red was the initial indication of successful nanoparticle synthesis. UV-Visible spectroscopy was employed to further validate this, revealing a characteristic absorption peak at 540 nm. The crystalline nature and size distribution of the Au-NPs were analysed using SAED and DLS, respectively. TEM verified the spherical shape of the Au-NPs. FTIR was employed to identify functional groups, including hydroxyl, amine, and alkyl groups, that are associated with biomolecules on the nanoparticle surface. The mitochondrial membrane was significantly disrupted by the Au-NPs, which exhibited significant anti-cancer activity against A549 lung cancer cells. The pro-apoptotic effects of the nanoparticles were assessed by evaluating intracellular ROS levels and caspase enzyme activity, with additional confirmation provided by DAPI staining. In A549 cells treated with Au-NPs, apoptosis induction was indicated by increased ROS generation and increased activities of caspases 3 and 9. These results emphasize the significant anti-cancer potential of Au-NPs, notably against A549 lung cancer cells, indicating their potential as a therapeutic option for lung cancer treatment.

## Data Availability

The raw data supporting the conclusions of this article will be made available by the authors, without undue reservation.
