# Supplementary material for: Moringa oleifera mediated green synthesis of gold nanoparticles and their anti-cancer activity against A549 cell line of lung cancer through ROS/ mitochondrial damage
Source: Front Chem. 2025 Mar 5;13:1521089. doi: 10.3389/fchem.2025.1521089 (PMC11920177; doi:10.3389/fchem.2025.1521089)
Supplement: Supplementary file 1 [file DataSheet1.docx]

**Supplementary information**

**Figure S1:**

**
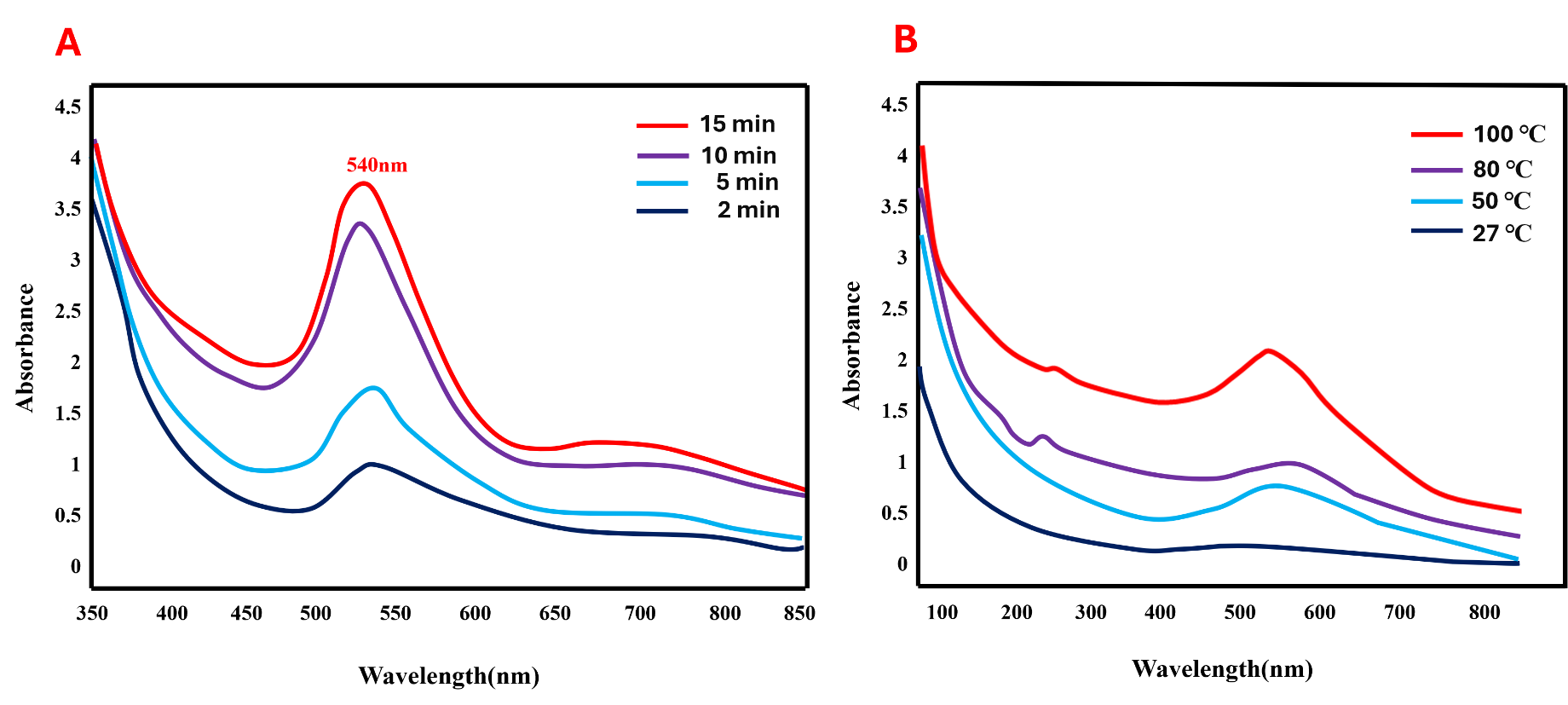
**

***Fig S1:*** *UV spectra of Au-NPs synthesised using M. oleifera at different temperatures.*

**Figure S2:**

**
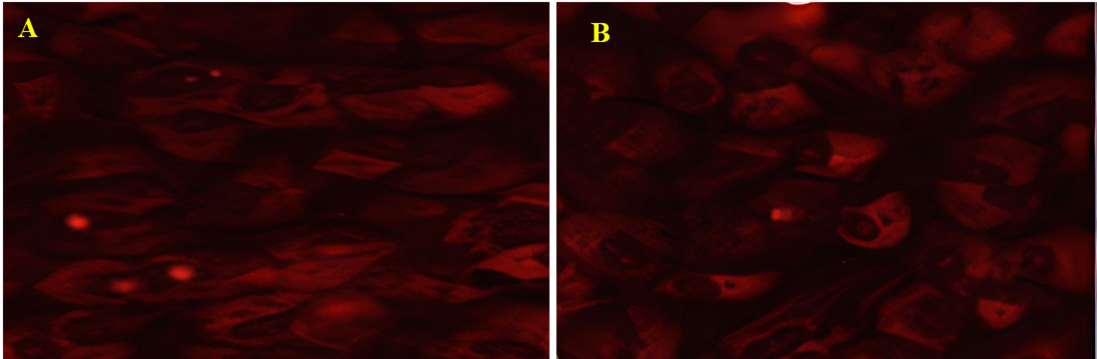
**

**Fig S2:** Intracellular mitochondrial membrane damage (A) and control (B) cells of biosynthesized Ag NPs against A549 cell line
